# Diversity of rickettsiae in ticks (Acari: Ixodidae) collected from wild vertebrates in part of the Amazon, Cerrado, and Pantanal biomes in Brazil

**DOI:** 10.1590/S1984-29612023059

**Published:** 2023-10-13

**Authors:** Anny Carolina Prati, Maerle Oliveira Maia, Thiago Fernandes Martins, Thaís Oliveira Morgado, Sandra Helena Ramiro Corrêa, Edson Junior Figueiredo Mendes, Rosa Helena dos Santos Ferraz, Jessica Rhaiza Mudrek, Christine Strüssmann, Dirceu Guilherme de Souza Ramos, Thiago Borges Fernandes Semedo, Make Kawatake Minetto, Daniel Moura de Aguiar, Richard Campos Pacheco, Andréia Lima Tomé Melo

**Affiliations:** 1 Programa de Pós-graduação em Biociência Animal, Universidade de Cuiabá - UNIC, Cuiabá, MT, Brasil; 2 Programa de Pós-graduação em Ciências Veterinárias - PPGVET, Faculdade de Medicina Veterinária - FAVET, Universidade Federal de Mato Grosso - UFMT, Cuiabá, MT, Brasil; 3 Departamento de Medicina Veterinária Preventiva e Saúde Animal, Faculdade de Medicina Veterinária e Zootecnia, Universidade de São Paulo - USP, São Paulo, SP, Brasil; 4 Instituto Pasteur, Secretaria de Estado da Saúde de São Paulo, São Paulo, SP, Brasil; 5 Programa de Pós-graduação em Zoologia, Instituto de Biociências, Universidade Federal de Mato Grosso - UFMT, Cuiabá, MT, Brasil; 6 Laboratório de Parasitologia e Análises Clínicas Veterinária, Programa de Pós-graduação em Biociência Animal, Unidade Acadêmica de Ciências Veterinárias, Universidade Federal de Jataí - UFJ, Jataí, GO, Brasil; 7 Departamento de Biologia, Faculdade de Ciências, Universidade do Porto, Porto, Portugal; 8 InBIO Laboratório Associado, Centro de Investigação em Biodiversidade e Recursos Genéticos - CIBIO, Universidade do Porto, Vairão, Portugal; 9 Programa BIOPOLIS em Genómica, Biodiversidade e Ordenamento do Território, CIBIO, de Vairão, Vairão, Portugal; 10 Instituto de Defesa Agropecuária do Estado de Mato Grosso - INDEA-MT, Cuiabá, MT, Brasil; 11 Laboratório de Parasitologia Veterinária e Doenças Parasitárias dos Animais Domésticos e Silvestres, Hospital Veterinário - HOVET, Faculdade de Medicina Veterinária - FAVET, Universidade Federal de Mato Grosso - UFMT, Cuiabá, MT, Brasil

**Keywords:** Wildlife, Ixodidae, Rickettsiae, tick-borne diseases, Animais selvagens, Ixodidae, Rickettsiae, doenças transmitidas por carrapatos

## Abstract

Ticks parasitizing 102 wild animals in the states of Mato Grosso and Goiás, Brazil were collected between 2015 and 2018. A total of 2338 ticks (865 males, 541 females, 823 nymphs, and 109 larvae) belonging to four genera (*Amblyomma*, *Dermacentor*, *Haemaphysalis*, and *Rhipicephalus*) and at least 21 species were identified. DNA extraction and a molecular survey for rickettsial agents were performed on 650 ticks. The results revealed parasitism by the following species: *Rickettsia amblyommatis* in *Amblyomma cajennense* s.s., *A. cajennense* s.l., *Amblyomma coelebs*, *Amblyomma humerale*, *Amblyomma longirostre*, *Amblyomma nodosum*, *Amblyomma scalpturatum*, *Amblyomma sculptum*, and *Amblyomma romitii*; *Rickettsia parkeri* in *Amblyomma nodosum*, *Amblyomma ovale*, *Amblyomma scalpturatum*, and *Amblyomma triste*; *Rickettsia rhipicephali* in *Haemaphysalis juxtakochi*; *Rickettsia* sp. in *A. cajennense* s.s., *A. nodosum*, and *A. sculptum*, and lastly, ‘*Candidatus* Rickettsia andeanae’ in *Amblyomma parvum* and *Rhipicephalus microplus*. This study expands the body of knowledge about tick parasitism among wild animals, including new data concerning tick-host associations, and provides information about the epidemiology of tick-borne pathogens in the Center-West region of Brazil.

## Resumo

Foram coletados carrapatos parasitando 102 animais silvestres nos estados de Mato Grosso e Goiás, Brasil, entre 2015 e 2018. Um total de 2338 carrapatos (865 machos, 541 fêmeas, 823 ninfas e 109 larvas) pertencentes a quatro gêneros (*Amblyomma*, *Dermacentor*, *Haemaphysalis* e *Rhipicephalus*) e pelo menos 21 espécies foram identificadas. A extração de DNA e pesquisa molecular para agentes riquétsiais foram realizadas em 650 carrapatos. Os resultados revelaram parasitismo pelas seguintes espécies: *Rickettsia amblyommatis* em *Amblyomma cajennense* s.s., *A. cajennense* s.l., *Amblyomma coelebs*, *Amblyomma humerale*, *Amblyomma longirostre*, *Amblyomma nodosum*, *Amblyomma scalpturatum*, *Amblyomma sculptum* e *Amblyomma romitii*; *Rickettsia parkeri* em *Amblyomma nodosum*, *Amblyomma ovale*, *Amblyomma scalpturatum* e *Amblyomma triste*; *Rickettsia rhipicephali* em *Haemaphysalis juxtakochi*; *Rickettsia* sp. em *A*. *cajennense* s.s., *A. nodosum* e *A. sculptum* e, por último, ‘*Candidatus* Rickettsia andeanae’ em *Amblyomma parvum* e *Rhipicephalus microplus*. Este estudo amplia o conhecimento sobre o parasitismo de carrapatos em animais silvestres, incluindo-se novos dados sobre associações carrapato-hospedeiro. Fornece, ainda, informações sobre a epidemiologia de patógenos transmitidos por carrapatos na região Centro-Oeste do Brasil.

## Introduction

Ticks (Acari: Argasidae and Ixodidae) are ectoparasitic arthropods of numerous animal species, while humans are accidental hosts, and one of the most important arthropod vectors of infectious diseases around the world (Anderson & Magnarelli, 2008; Nava et al., 2009; Nogueira et al., 2022). Several studies have focused on ticks and their associations with wildlife in the Central-West region of Brazil (e.g., Aragão, 1936; Aragão & Fonseca, 1961; Szabó et al., 2007; Martins et al., 2011, 2023; Soares et al., 2015; Witter et al., 2016; Colle et al., 2020; Serpa et al., 2021), and an understanding of aspects pertaining both to these ectoparasites and to the infectious agents they can transmit to their hosts during hematophagy is extremely important.

Rickettsiae (Rickettsiales: Rickettsiaceae) are small obligate intracellular gram-negative bacteria that infect invertebrate and vertebrate hosts worldwide with transmission related to ectoparasitic arthropods, mainly ticks (Dumler et al., 2001). Currently, the genus *Rickettsia* has been classified in the Spotted Fever Group (SFG), the Typhus Group (TG), the Transitional Group (TRG), the Bellii Group (BG), the Canadensis Group (CG), and several other basal groups (Weinert et al., 2009).

In Brazil, the main tick-borne disease is Brazilian Spotted Fever (BSF), caused by the bacterium *Rickettsia rickettsii*, which is responsible for a high mortality rate among infected humans (Oliveira et al., 2016). This zoonosis is transmitted by the ticks *Amblyomma sculptum*, the most important vector in Brazil (Labruna, 2009) in many parts of endemic areas in southeastern Brazil, including the states of São Paulo, Rio de Janeiro, Espírito Santo, and Minas Gerais; and *Amblyomma aureolatum*, a recognized vector within the metropolitan area of São Paulo municipality within the Atlantic rainforest mountain domain (Binder et al., 2021). Furthermore, another pathogenic rickettsial agent, known as *Rickettsia parkeri* strain Atlantic rainforest (Krawczak et al., 2016), transmitted mainly by adult *Amblyomma ovale* ticks (Szabó et al., 2013), has been associated with mild cases among humans in Brazil in the States of São Paulo (Spolidorio et al., 2010), Bahia (Silva et al., 2011), and Santa Catarina (Krawczak et al., 2016). Lastly, other species of the genus *Rickettsia* have been identified in the SFG, namely *R. amblyommatis* and *R. rhipicephali*, infecting ticks, but pathogenicity in humans is still unknown (Parola et al., 2013).

Thus, the occurrence of tick species among varied wild hosts in different biomes (Amazon, Cerrado, and Pantanal), where domestic animals and humans also inhabit, considering the importance of ticks and rickettsial diseases for public health, reinforces the need for research focused on this subject. In view of the importance of the tick-host association and research on rickettsial infection in ticks, this investigation focused on the molecular detection of rickettsiae in ticks collected from free-living or captive wild animals in the Central-West region of Brazil.

## Materials and Methods

**Figure 1:**
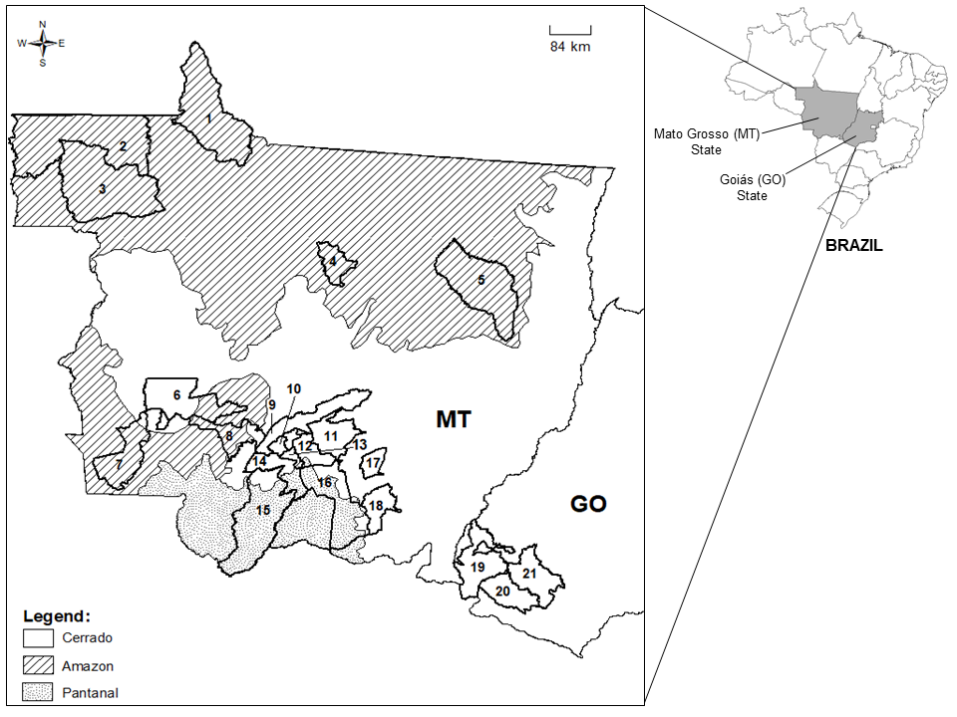
Municipalities where the ticks examined in this study were collected between 2015 and 2018 in the states of Mato Grosso (MT): 1. Apiacás; 2. Colniza; 3. Aripuanã; 4. Sinop; 5. Querência; 6. Tangará da Serra; 7. Pontes e Lacerda; 8. Barra do Bugres; 9. Rosário Oeste; 10. Jangada; 11. Chapada dos Guimarães; 12. Cuiabá; 13. Várzea Grande; 14. Nossa Senhora do Livramento; 15. Poconé; 16. Santo Antônio de Leverger; 17. Dom Aquino; 18. Rondonópolis, and Goiás (GO): 19. Mineiros; 20. Serranópolis; 21. Jataí.

Ticks were collected between 2015 and 2018 from free-living and captive wild animals in the states of Mato Grosso (MT) in part of the Amazon, Cerrado, and Pantanal biomes, and Goiás (GO) in the Cerrado biome, both located in the Central-West region of Brazil (Figure 1). Samples were obtained from road-killed wild animals and from wild animals (n= 94) treated at the Veterinary Hospital of the Federal University of Mato Grosso (UFMT) (Cuiabá, MT), and from wild animals (n= 8) sent to the Wild Animal Screening Center (CETAS) of the Brazilian Institute of Environment and Renewable Natural Resources (IBAMA) in Goiânia, GO (Table 1). Ticks were preserved in isopropyl alcohol for taxonomic identification and DNA extraction. Adult ticks were identified to species level, as described by Barros-Battesti et al. (2006), Martins et al. (2016), and Nava et al. (2018), while *Amblyomma* and *Haemaphysalis* nymphs were identified morphologically as specified by Martins et al. (2010, 2016) and Nava et al. (2017), respectively. The larvae of the genus *Amblyomma* could not be morphologically identified to the species level because there is insufficient literature available, then the larvae were retained as *Amblyomma* sp. according to Vieira et al. (2004), Barros-Battesti et al. (2006), and Nava et al. (2017). The larva of the genus *Dermacentor* was identified morphologically as described by Clifford & Anastos (1960), thus, the larva identified in the genus *Dermacentor* was considered to be *D. nitens*. Finally, adults of *Rhipicephalus linnaei* were identified based on Šlapeta et al. (2022). Tick specimens were deposited in the Tick Collection *Coleção de Carrapatos da Universidade Federal de Mato Grosso* at the Federal University of Mato Grosso, in Cuiabá, MT, under the following accession numbers: LDPP-UFMT/N.124-127; 129; 133; 135-137; 139-140; 142-144; 148-150; 155; 157-158; 160-163; 165-174; 176-179; 181; 185; 187-188; 190-195; 199-200; 203; 210; 214-217; 220; 224-227; 229; 231-239; 242; 244-247; 255-256; 260-266; 270-277; 283; 287-291; 294; 296-297 and 303-306.

**Table 1:** Ticks (M: male; F: female; N: nymph; L: larva) parasitizing free-living (FL) and captive (C) wild animals, collected between 2015 and 2018, in the states of Mato Grosso (MT) and Goiás (GO), in the Central-West region of Brazil.

**Hosts (n. of specimens when >1)**	**Origin (Year)**	**Municipality (State)**	**Species (n. of collected ticks)**
**ORDER CARIAMIFORMES**			
*Cariama cristata* (2)	FL (2017)	Cuiabá (MT)	*Amblyomma sculptum* (4M, 6F, 24N)
			
**ORDER ANURA**			
*Rhinella diptycha* (2)	FL (2017)	Cuiabá (MT)	*Amblyomma rotundatum* (7F, 14N)
*R. diptycha* (2)	FL (2017)	Jataí (GO)	*A. rotundatum* (5F)
			
**ORDER TESTUDINES**			
*Chelonoidis carbonarius*	FL (2016)	Cuiabá (MT)	*A. rotundatum* (2F)
*Chelonoidis denticulatus*	FL (2017)	Apiacás (MT)	*Amblyomma cajennense* sensu stricto (s.s.) (1F), *Amblyomma humerale* (9M, 1N), *A. rotundatum* (1F)
*C. denticulatus*	FL (2017)	Cuiabá (MT)	*A. rotundatum* (3F)
*Phrynops geoffroanus*	FL (2016)	Cuiabá (MT)	*A. sculptum* (2M, 1F)
			
**ORDER CROCODYLIA**			
*Caiman yacare*	FL (2015)	Cuiabá (MT)	*Amblyomma dissimile* (1M, 2F)
*Paleosuchus palpebrosus* (2)	FL (2016)	Cuiabá (MT)	*A. dissimile* (1F), *A. rotundatum* (1F)
			
**ORDER SQUAMATA**			
**Suborder Sauria**			
*Iguana iguana*	FL (2015)	Cuiabá (MT)	*A. dissimile* (19M, 23F, 8N), *Amblyomma* sp. (1L)
*I. iguana* (2)	FL (2016)	Cuiabá (MT)	*A. dissimile* (7M, 1F)
*I. iguana*	FL (2017)	Cuiabá (MT)	*A. dissimile* (2M, 1F)
*I. iguana* (2)	FL (2018)	Cuiabá (MT)	*A. dissimile* (17M, 5F, 5N), *Amblyomma* sp. (2L)
*Tupinambis* sp.	FL (2015)	Poconé (MT)	*A. rotundatum* (6N), *Amblyomma* sp. (4L)
			
**Suborder Serpentes**			
*Boa constrictor*	FL (2015)	Cuiabá (MT)	*A. dissimile* (84M, 50F, 133N)
*B. constrictor*	FL (2017)	Jataí (GO)	*A. rotundatum* (3F)
*B. constrictor* (2)	FL (2018)	Cuiabá (MT)	*A. dissimile* (5M, 2F)
*B. constrictor*	FL (2018)	Poconé (MT)	*A. dissimile* (16M, 5N)
*Bothrops mattogrossensis*	FL (2016)	Poconé (MT)	*A. dissimile* (2F)
*Eunectes notaeus*	FL (2015)	Poconé (MT)	*A. dissimile* (3M, 2F)
			
**ORDER ARTIODACTYLA**			
*Mazama americana*	FL (2016)	Colniza (MT)	*A. cajennense* s.s. (1F, 22N), *Amblyomma oblongoguttatum* (1F), *Haemaphysalis juxtakochi* (3F, 1N)
*Subulo gouazoubira*	FL (2016)	Cuiabá (MT)	*A. sculptum* (22N^a^)
*S. gouazoubira*	FL (2016)	Várzea Grande (MT)	*Rhipicephalus microplus* (2F)
*S. gouazoubira*	FL (2016)	Cuiabá (MT)	*R. microplus* (2M, 1F)
*S. gouazoubira*	FL (2017)	Cuiabá (MT)	*A. sculptum* (5N), *R. microplus* (19M, 14F, 4N)
*S. gouazoubira*	FL (2018)	Cuiabá (MT)	*Amblyomma parvum* (5M), *R. microplus* (4M, 2F, 2N)
*S. gouazoubira*	FL (2018)	Poconé (MT)	*R. microplus* (5M, 2F, 4N)
*Tayassu pecari*	FL (2017)	Apiacás (MT)	*A. cajennense* s.s. (2M, 2F), *Amblyomma naponense* (1F), *A. oblongoguttatum* (1F), *Amblyomma scalpturatum* (4N)
			
**ORDER CARNIVORA**			
*Cerdocyon thous*	FL (2018)	Cuiabá (MT)	*A. ovale* (1M, 1F)
*C*. *thous* (3)	FL (2018)	Poconé (MT)	*Amblyomma ovale* (5M), *A. sculptum* (30N), *Amblyomma* sp. (8L), *R. microplus* (1N)
*Chrysocyon brachyurus*	FL (2017)	Serranópolis (GO)	*A. ovale* (2F), *Dermacentor nitens* (1L)
*C. brachyurus*	FL (2018)	Serranópolis (GO)	*A. ovale* (1M, 2F), *Amblyomma* sp. (1L)
*Leopardus pardalis*	FL (2016)	Barra do Bugres (MT)	*Amblyomma oblongoguttatum* (1N^b^), *A. sculptum* (3N, 15L^c^), *Amblyomma* sp. (1L)
*Nasua nasua*	FL (2018)	Cuiabá (MT)	*A. ovale* (1M), *A. sculptum* (8N), *Amblyomma* sp. (7L)
*N. nasua*	FL (2018)	Poconé (MT)	*A. ovale* (1M), *A. sculptum* (37N), *Amblyomma* sp. (2L)
*Procyon cancrivorus*	FL (2018)	Poconé (MT)	*A. ovale* (2F), *A. sculptum* (3N)
*Puma concolor*	FL (2017)	Colniza (MT)	*A. cajennense* s.s. (8N)
*P. concolor*	FL (2017)	Tangará da Serra (MT)	*A. cajennense* s.s. (1F), *A. cajennense* s.l. (1M, 3N), *Amblyomma coelebs* (1M), *A. ovale* (1M), *Amblyomma* sp. (14L), *R. microplus* (1N)
			
**ORDER CINGULATA**			
*Euphractus sexcinctus*	FL (2017)	Colniza (MT)	*A. cajennense* s.s. (2N), *A. coelebs* (6N), *Amblyomma* sp. (3L)
			
**ORDER DIDELPHIMORPHIA**			
*Didelphis marsupialis*	FL (2017)	Aripuanã (MT)	*A. coelebs* (6N), *Amblyomma humerale* (1N), *Amblyomma* sp. (16L)
			
**ORDER PERISSODACTYLA**			
*Tapirus terrestris*	FL (2016)	Chapada dos Guimarães (MT)	*A. coelebs* (2F), *A. sculpum* (39M, 20F, 92N^d^)
*T*. *terrestris*	FL (2016)	Dom Aquino (MT)	*A. coelebs* (1M, 3F), *A. oblongoguttatum* (1M), *A. sculptum* (23M, 8F, 2N), *R. microplus* (3F)
*T. terrestris*	FL (2016)	Pontes e Lacerda (MT)	*A. coelebs* (1F), *A. cajennense* s.l. (10M)
*T. terrestris*	FL (2016)	Sinop (MT)	*A. cajennense* s.s. (143M, 20F, 14N), *A. coelebs* (7M, 1F), *A. ovale* (1M)
*T. terrestris*	FL (2017)	Chapada dos Guimarães (MT)	*A. coelebs* (2M), *A. sculptum* (5M, 13F, 1N)
*T. terrestris* (3)	FL (2017)	Colniza (MT)	*A. cajennense* s.s. (2M, 9F, 3N), *A. coelebs* (3M, 4F), *A. oblongoguttatum* (1M, 2F), *A. scalpturatum* (3M, 14F)
*T. terrestris*	FL (2017)	Poconé (MT)	*A. sculptum* (34M, 7F)
*T. terrestris* (2)	FL (2017)	Pontes e Lacerda (MT)	*A. coelebs* (13M, 9F, 1N), *A. oblongoguttatum* (1M, 3F), *Amblyomma scalpturatum* (39M, 14F), *A. cajennense* s.l. (29M) *A. sculptum* (31F, 2N), *R. microplus* (2F)
*T. terrestris*	FL (2017)	Rondonópolis (MT)	*A. coelebs* (4M, 1F), *A. ovale* (1M), *A. triste* (5M, 6F), *A. sculptum* (60M, 12F, 1N), *R. microplus* (5F, 1N)
*T. terrestris*	FL (2017)	Querência (MT)	*R. microplus* (1M, 6F)
*T. terrestris*	FL (2018)	Colniza (MT)	*A. coelebs* (2M), *A. cajennense* s.s. (1M, 4F, 1N), *A. scalpturatum* (1F)
			
**ORDER PILOSA**			
*Myrmecophaga tridactyla*	FL (2015)	Cuiabá (MT)	*Amblyomma nodosum* (4M, 5F), *A. parvum* (1F), *A. sculptum* (6M, 7F, 54N), *Amblyomma* sp. (8L)
*M. tridactyla*	FL (2016)	Barra do Bugres (MT)	*A. nodosum* (3F), *A. sculptum* (1N)
*M. tridactyla* (2)	C (2016)	Cuiabá (MT)	*A. sculptum* (3M, 1F, 7N^e^)
*M. tridactyla*	FL (2016)	Mineiros (GO)	*A. nodosum* (4M), *A. sculptum* (1F)
*M. tridactyla*	FL (2016)	Rosário Oeste (MT)	*A. nodosum* (1M, 1F), *A. sculptum* (8M, 2F, 23N^f^)
*M. tridactyla*	FL (2016)	Tangará da Serra (MT)	*A. nodosum* (1M, 5F), *A. cajennense* s.l. (12M, 20N^g^), *A. cajennense* s.s. (3N^h^), *A. scalpturatum* (2M), *A. sculptum* (10F, 5N^i^)
*M. tridactyla*	FL (2017)	Poconé (MT)	*A. sculptum* (7M, 1F)
*M. tridactyla*	FL (2017)	Várzea Grande (MT)	*A. sculptum* (3M, 2F, 1N)
*M. tridactyla* (2)	FL (2018)	Cuiabá (MT)	*A. nodosum* (2M), *A. sculptum* (17N), *Amblyomma* sp. (3L)
*M. tridactyla*	FL (2018)	Santo Antônio de Leverger (MT)	*A. nodosum* (4M, 3F), *A. sculptum* (12N), *Amblyomma* sp. (8L)
*Tamandua tetradactyla*	FL (2015)	Cuiabá (MT)	*A. nodosum* (2M)
*T. tetradactyla*	FL (2016)	Cuiabá (MT)	*A. nodosum* (2M)
*T. tetradactyla*	FL (2016)	Jangada (MT)	*A. nodosum* (1M, 2F)
*T. tetradactyla* (2)	FL (2016)	Mineiros (GO)	*A. nodosum* (10M, 4F)
*T. tetradactyla*	FL (2016)	Várzea Grande (MT)	*A. nodosum* (2F)
*T. tetradactyla*	FL (2017)	Chapada dos Guimarães (MT)	*Amblyomma calcaratum* (23M), *A. coelebs* (1N), *A. nodosum* (1M), *A. sculptum* (1F), *Amblyomma* sp. (7L)
*T. tetradactyla*	FL (2017)	Nossa Senhora do Livramento (MT)	*A. nodosum* (4M, 4F), *A. sculptum* (1F, 1N)
*T. tetradactyla* (2)	FL (2017)	Poconé (MT)	*A. nodosum* (4M, 4F), *A. sculptum* (2M, 2F, 1N)
*T. tetradactyla*	FL (2017)	Santo Antônio de Leverger (MT)	*A. nodosum* (8M, 2F)
*T. tetradactyla* (4)	FL (2018)	Poconé (MT)	*A. nodosum* (35M, 24F), *A. sculptum* (3F, 19N)
			
**ORDER RODENTIA**			
*Coendou longicaudatus*	FL (2015)	Chapada dos Guimarães (MT)	*Amblyomma longirostre* (8M, 1F)
*Cuniculus paca*	FL (2015)	Cuiabá (MT)	*A. naponense* (2N^j^)
*Holochilus sciureus*	FL (2017)	Poconé (MT)	*A. triste* (6N)
*Hydrochoerus hydrochaeris*	C (2016)	Cuiabá (MT)	*A. sculptum* (7M, 4F)
*H. hydrochaeris* (2)	FL (2016)	Cuiabá (MT)	*Amblyomma dubitatum* (2N^l^), *A. sculptum* (1M, 7F, 36N^m^)
*H. hydrochaeris*	FL (2016)	Várzea Grande (MT)	*A. dubitatum* (1M; 11F, 3N^n^), *A. sculptum* (4M, 12F, 91N^o^), *Amblyomma* sp. (8L)
*H. hydrochaeris*	FL (2017)	Colniza (MT)	*A. cajennense* s.l. (1M), *Amblyomma romitii* (8M, 12F, 4N)
*H. hydrochaeris*	FL (2017)	Cuiabá (MT)	*A. dubitatum* (2N), *A. sculptum* (7M, 4F, 19N)
*H. hydrochaeris*	FL (2017)	Poconé (MT)	*A. sculptum* (2M, 4F, 3N)
*H. hydrochaeris*	FL (2017)	Pontes e Lacerda (MT)	*A. dubitatum* (6M, 28F), *A. sculptum* (2N)
*H. hydrochaeris*	FL (2017)	Várzea Grande (MT)	*A. dubitatum* (1M, 3F), *A. sculptum* (6M, 4F), *Rhipicephalus linnaei* (15M, 7F)

a Reared from nymphs (n= 22) at the laboratory conditions which molted to the adult stage (n= 11 males, 11 females);

b Reared from nymph (n= 1) at the laboratory conditions which molted to the adult stage (n= 1 male);

c Reared from larvae (n= 15) at the laboratory conditions which molted to the nymph stage (n= 15), thus identified at species level;

d Reared from nymphs (n= 30) at the laboratory conditions which molted to the adult stage (n= 11M, 19F);

e Reared from nymphs (n= 6) at the laboratory conditions which molted to the adult stage (n= 2 males, 4 females);

f Reared from nymphs (n= 23) at the laboratory conditions which molted to the adult stage (n= 11 males, 11 females);

g Reared from nymphs (n= 20) at the laboratory conditions which molted to the adult stage (n= 8 males);

h Reared from nymphs (n= 3) at the laboratory conditions which molted to the adult stage (n= 3 females);

i Reared from nymphs (n= 5) at the laboratory conditions which molted to the adult stage (n= 5 females);

j Reared from nymphs (n= 2) at the laboratory conditions which molted to the adult stage (n= 2 females);

l Reared from nymph (n= 1) at the laboratory conditions which molted to the adult stage (n= 1 female);

m Reared from nymphs (n= 36) at the laboratory conditions which molted to the adult stage (n= 11 males, 11 females);

n Reared from nymphs (n= 3) at the laboratory conditions which molted to the adult stage (n= 1 male);

o Reared from nymphs (n= 91) at the laboratory conditions which molted to the adult stage (n= 26 males, 45 females).

In the molecular screening for the detection of rickettsial agents, we attempted to choose all tick species identified, which had more than one specimen per host, including the largest number of vertebrate hosts among each order of animals sampled. Therefore, some whole ticks were subjected to DNA extraction using the guanidine isothiocyanate protocol, being placed in a 1.5-mL microtube containing 150 µL of TE buffer (Tris HCl 10 mmol/L, EDTA 1 mmol/L, pH 7.4) and homogenized by using a sterile pipette tip as described by Sangioni et al. (2005), and to amplification by a conventional polymerase chain reaction (cPCR). To this end, an initial cPCR was performed using CS-78 and CS-323 primers, which target a fragment of 401 bp (base pairs) of the citrate synthase (*gltA*) gene common to all *Rickettsia* species, according to Labruna et al. (2004). Positive samples were further tested by another cPCR protocol using Rr190.70p and Rr190.602n primers, which amplify a ~530 bp fragment of the 190-kDa outer membrane protein gene (*ompA*) found only in *Rickettsia* of the SFG (Regnery et al., 1991). Negative (nuclease-free water) and positive controls (*R. rickettsii* DNA) were included in each of these reactions.

*Rickettsia* amplicons of the expected size were purified using the Illustra GFX PCR DNA and Gel Band Purification Kit (GE Healthcare, Chicago, Illinois) and sent for sequencing at the company ACTGene (Porto Alegre, RS, Brazil) with the same primers used in the cPCR. To evaluate the quality of the sequences, electropherograms were verified with CLC Genomics Workbench software (Qiagen^®^). All the sequences obtained were then analyzed using the Basic Local Alignment Search Tool (BLAST; Altschul et al., 1990) to determine the closest identities with congeneric organisms available in GenBank.

## Results

The ticks collected in this study were taken from 102 wild animals, three of which lived in captivity and 99 were free-living individuals, distributed among 12 orders in four classes, as follows: four amphibian species, 21 reptiles, two birds, and 75 mammals. These animals were parasitized by 2338 ticks (865 males, 541 females, 823 nymphs, and 109 larvae) belonging to four genera and at least 21 species: 1239 ticks of the *Amblyomma cajennense* complex [*A. sculptum* (223 males, 164 females, 522 nymphs, and 15 larvae), *Amblyomma cajennense* s.s. (148 males, 38 females, and 53 nymphs), *A. cajennense* s.l. (53 males, and 23 nymphs)], 394 *Amblyomma dissimile* (154 males, 89 females, and 151 nymphs), 142 *Amblyomma nodosum* (83 males and 59 females), 81 *Rhipicephalus microplus* (31 males, 37 females, and 13 nymphs), 77 *Amblyomma scalpturatum* (44 males, 29 females, and 4 nymphs), 68 *Amblyomma coelebs* (33 males, 21 females, and 14 nymphs), 57 *Amblyomma dubitatum* (8 males, 42 females, and 7 nymphs), 37 *Amblyomma rotundatum* (17 females and 20 nymphs), 24 *Amblyomma romitii* (8 males, 12 females, and 4 nymphs), 23 *Amblyomma calcaratum* (23 males), 22 *R. linnaei* (15 males and 7 females), 19 *A. ovale* (12 males and 7 females), 17 *Amblyomma triste* (5 males, 6 females, and 6 nymphs), 11 *Amblyomma humerale* (9 males and 2 nymphs), 11 *Amblyomma oblongoguttatum* (3 males, 7 females, and 1 nymph), 9 *Amblyomma longirostre* (8 males and 1 female), 6 *Amblyomma parvum* (5 males and 1 female), 3 *Amblyomma naponense* (1 female and 2 nymphs), and 4 *Haemaphysalis juxtakochi* (3 females and 1 nymph). In addition, one larva of *D. nitens* was identified, but 93 larvae could not be identified at the species level and were therefore classified as *Amblyomma* sp. (Table 1).

A total of 650 DNA-extracted random samples were tested individually (430 adults) and in pools (40 pools of 5 nymphs and two pools of 10 larvae) to screen for rickettsial agents by cPCR targeting the rickettsial *gltA* gene. These samples were as follows: *Amblyomma* spp. (20 larvae), *A. cajennense* s.s. (5 males, 1 female, and 30 nymphs), *A. cajennense* s.l. (15 males), *A. calcaratum* (20 males), *A. coelebs* (13 males, 14 females, and 5 nymphs), *A. dissimile* (43 males, 10 females, and 20 nymphs), *A. dubitatum* (5 males and 5 females), *A. humerale* (8 males), *A. longirostre* (4 males), *A. naponense* (1 female), *A. nodosum* (50 males and 19 females), *A. oblongoguttatum* (2 females), *A. ovale* (5 males and 2 females), *A. parvum* (4 males), *A. romitii* (7 males and 11 females), *A. rotundatum* (9 females and 15 nymphs), *A. scalpturatum* (40 males and 25 females), *A. sculptum* (32 males, 36 females, and 125 nymphs), *A. triste* (4 males, 3 females, and 5 nymphs), *H. juxtakochi* (3 females), and *R. microplus* (27 males and 7 females), as detailed in Table 2.

**Table 2:** Results of molecular tests on ticks (M: adult male; F: adult female; N: nymph; L: larva) collected from free-living and captive wild animals between 2015 and 2018, in the states of Mato Grosso (MT) and Goiás (GO), in the Central-West region of Brazil.

**Hosts**	**Municipalities**	**Ticks (No. number of specimens)**	**No. infected/**	**Closest GenBank identity (gene: accession number)**
			**No. tested (%)**	
**ORDER CARIAMIFORMES**				
*Cariama cristata*	Cuiabá (MT)	*Amblyomma sculptum* (2M, 2F, 20N)	1/24 (4.16^a^)	99% *Rickettsia amblyommatis* (*omp*A: MH818422)
				
**ORDER ANURA**				
*Rhinella diptycha*	Cuiabá (MT), Jataí (GO)	*Amblyomma rotundatum* (3F, 10N)	0/13	
				
**ORDER TESTUDINES**				
*Chelonoidis carbonarius*	Cuiabá (MT)	*A. rotundatum* (1F)	0/1	
*Chelonoidis denticulatus*	Apiacás (MT), Cuiabá (MT)	*Amblyomma humerale* (8M)	2/8 (25)	100% *R*. *amblyommatis* (*omp*A: MF188914)
	
		*A. rotundatum* (2F)	0/2	
*Phrynops geoffroanus*	Cuiabá (MT)	*A. sculptum* (1M)	0/1	
				
**ORDER CROCODYLIA**				
*Caiman yacare*	Cuiabá (MT)	*Amblyomma dissimile* (1F)	0/1	
*Paleosuchus palpebrosus*	Cuiabá (MT)	*A. dissimile* (1F)	0/1	
		*A. rotundatum* (1F)	0/1	
				
**ORDER SQUAMATA**				
**Suborder Sauria**				
*Iguana iguana*	Cuiabá (MT)	*A. dissimile* (20M, 1F, 10N)	0/31	
*Tupinambis* sp.	Poconé (MT)	*A. rotundatum* (5N)	0/5	
				
**Suborder Serpentes**				
*Boa constrictor*	Cuiabá (MT), Jataí (GO)	*A. dissimile* (22M, 5F, 10N)	0/37	
		*A. rotundatum* (2F)	0/2	
*Bothrops mattogrossensis*	Poconé (MT)	*A. dissimile* (1F)	0/1	
*Eunectes notaeus*	Poconé (MT)	*A. dissimile* (1M, 1F)	0/2	
				
**ORDER ARTIODACTYLA**				
*Mazama americana*	Colniza (MT)	*Amblyomma cajennense* s.s. (1F, 20N)	5/21 (23.80^a^)	100% *Rickettsia* sp. (*gltA*: KY753118, MK441839, MK720995, MK720994)
				100% *R. amblyommatis* (*omp*A: MN336348)
				100% *Rickettsia rhipicephali* (*omp*A: KX434736)
		*Haemaphysalis juxtakochi* (3F)	2/3 (66.66)	
		*Amblyomma oblongoguttatum* (1F)	0/1	
		
*Subulo gouazoubira*	Cuiabá (MT), Poconé (MT), Várzea Grande (MT)	*Amblyomma parvum* (4M)	2/4 (50)	100% ‘*Candidatus* Rickettsia andeanae’ (*gltA*: MG887826)
		*Rhipicephalus microplus* (27M, 1F)	2/28 (7.14)	100% ‘*Ca.* Rickettsia andeanae’ (*gltA*: MG887826)
		*A. sculptum* (1M, 1F, 5N)	0/7	
*Tayassu pecari*	Apiacás (MT)	*A. cajennense* s.s. (1M)	0/1	
				
**ORDER CARNIVORA**				
*Cerdocyon thous*	Cuiabá (MT), Poconé (MT)	*Amblyomma ovale* (3M)	0/3	
		*A. sculptum* (20N)	0/20	
*Chrysocyon brachyurus*	Serranópolis (GO)	*A. ovale* (1M, 1F)	1/2 (50)	100% *Rickettsia parkeri* strain Atlantic rainforest (*omp*A: MK801772)
*Leopardus pardalis*	Barra do Bugres (MT)	*A. sculptum* (5N)	0/5	
*Nasua nasua*	Cuiabá (MT), Poconé (MT)	*A. sculptum* (15N)	0/15	
*Procyon cancrivorus*	Poconé (MT)	*A. ovale* (1F)	1/1 (100)	100% *R. parkeri* strain Atlantic rainforest (*omp*A: MK801772)
*Puma concolor*	Colniza (MT), Tangará da Serra (MT)	*A. cajennense* s.s. (5N)	0/5	
		*Amblyomma* sp. (10L)	0/10	
				
**ORDER CINGULATA**				
*Euphractus sexcinctus*	Colniza (MT)	*A. coelebs* (5N)	0/5	
				
**ORDER DIDELPHIMORPHIA**				
*Didelphis marsupialis*	Aripuanã (MT)	*Amblyomma* sp. (10L)	0/10	
				
**ORDER PERISSODACTYLA**				
*Tapirus terrestris*	Chapada dos Guimarães (MT), Colniza (MT), Dom Aquino (MT), Poconé (MT), Pontes e Lacerda (MT), Querência (MT), Rondonópolis (MT), Sinop (MT)	*A. coelebs* (13M, 14F)	6/27 (22.22)	100% *R*. *amblyommatis* (*omp*A: MW147461)
		*Amblyomma scalpturatum* (39M, 25F)	4/64 (6.25)	100% *R*. *amblyommatis* (*omp*A: MW147461)
				100% *R. parkeri* strain NOD (*omp*A: MK522487, KY008394, KP987310, KM262193, EU567180)
				100% *R*. *amblyommatis* (*omp*A: MW147461)
		*A. sculptum* (10M, 19F, 5N)	4/34 (11.76)	100% *R*. *amblyommatis* (*omp*A: MW147461)
		*A. cajennense* s.l. (15M)	5/15 (33.33)	100% *R*. *amblyommatis* (*omp*A: MG787411, MN336348)
		*A. cajennense* s. s. (4M, 5N)	4/9 (44.44^a^)	
		*A. oblongoguttatum* (1F)	0/1	
		*A. ovale* (1M)	0/1	100% *R. parkeri* sensu stricto (*ompA*: MG574938)
		*A. triste* (4M, 3F)	1/7 (14.28)	
		*R. microplus* (6F)	0/6	
**ORDER PILOSA**				
*Myrmecophaga tridactyla*	Barra do Bugres (MT), Cuiabá (MT), Mineiros (GO), Poconé (MT), Rosário Oeste (MT), Santo Antônio de Leverger (MT), Tangará da Serra (MT), Várzea Grande (MT)	*Amblyomma nodosum* (10M, 6F)	2/16 (12.5)	100% *R*. *amblyommatis* (*omp*A: MW147461)
				100% *R. parkeri* strain NOD (*omp*A: MK522487, KY008394, KP987310, KM262193, EU567180)
				100% *Rickettsia* sp. (*gltA*: KY753118, MK441839, MK720995, MK720994)
		*A. sculptum* (7M, 3F, 25N)	1/35 (2.85^a^)	
		*A. scalpturatum* (1M)	0/1	
		
*Tamandua tetradactyla*	Chapada dos Guimarães (MT), Cuiabá (MT), Jangada (MT), Mineiros (GO), Nossa Senhora do Livramento (MT), Poconé (MT), Santo Antônio de Leverger (MT), Várzea Grande (MT)	*A. nodosum* (40M, 13F)	8/53 (15.09)	100% *Rickettsia* sp. (*gltA*: KY753118, MK441839, MK720995, MK720994)
				100% *R. parkeri* strain NOD (*omp*A: MK522487, KY008394, KP987310, KM262193, EU567180)
		
		
		*Amblyomma calcaratum* (20M)	0/20	
		*A. sculptum* (1M, 10N)	0/11	
				
**ORDER RODENTIA**				
*Coendou longicaudatus*	Chapada dos Guimarães (MT)	*Amblyomma longirostre* (4M)	1/4 (25)	100% *R. amblyommatis* (*omp*A: MG787411)
*Cuniculus paca*	Cuiabá (MT)	*A. naponense* (1F)	0/1	
*Hydrochoerus hydrochaeris*	Colniza (MT), Cuiabá (MT), Poconé (MT), Pontes e Lacerda (MT), Várzea Grande (MT)	*A. sculptum* (10M, 11F, 20N)	1/41 (2.43^a^)	100% *R. amblyommatis* (*gltA*: MH257786)
		*Amblyomma romitii* (7M, 11F)	1/18 (5.55)	100% *R. amblyommatis* (*gltA*: MH257786)
		*A. dubitatum* (5M, 5F)	0/10	
*Holochilus sciureus*	Poconé (MT)	*A. triste* (5N)	0/5	
**TOTAL**		**650 (282 males, 148 females, 200 nymphs, and 20 larvae)**	**54/650 (8.30** ^a^ **)**	

a Results refer to minimal infection rate because PCR-positive ticks included a pool of 5 nymphs or 10 larvae.

Among the 650 DNA-extracted samples from ticks evaluated by cPCR targeting the *gltA* gene, at least 54 (8.30%) were found to contain rickettsial DNA through the *gltA*-cPCR (Table 2). Furthermore, 43 of these *gltA*-PCR positive samples yielded amplicons after the *ompA*-PCR assay. Overall, 30 DNA sequences were generated, involving the following tick species: *A. cajennense* s.s. (1 adult individual and three nymphal pools), *A. coelebs* (4 adult individuals), *A. cajennense* s.l. (1 adult individual), *A. humerale* (1 adult individual), *A. longirostre* (1 adult individual), *A. nodosum* (7 adult individuals), *A. ovale* (2 adult individuals), *A. parvum* (1 adult individual), *A. romitii* (1 adult individual), *A. scalpturatum* (4 adult individuals), *A. sculptum* (1 adult individual and 3 nymphal pools), *A. triste* (1 adult individual), *H. juxtakochi* (1 adult individual) and *R. microplus* (1 adult individual).

Molecular screening for rickettsial agents and sequences obtained from *A. cajennense* s.s. (1 adult individual and two nymphal pools), *A. cajennense* s.l. (1 adult individual), *A. coelebs* (4 adult individuals), *A. humerale* (1 adult individual), *A. longirostre* (1 adult individual), *A. nodosum* (1 adult individual), *A. scalpturatum* (3 adult individuals), and *A. sculptum* (1 adult individual and one nymphal pool) showed five different haplotypes corresponding to sequences of *R. amblyommatis* (GeneBank accession numbers MH818422, MF188914, MW147461, MN336348, and MG787411), with a similarity of 99% (487/488 bp) to 100% (440/440 bp - 488/488 bp). Furthermore, partial sequences showed a 100% (447bp - 491bp) match to *R. parkeri* strains (MG574938, MK801772, MK522487) obtained from *A. scalpturatum* (1 adult individual), *A. nodosum* (4 adult individuals), *A. ovale* (2 adult individuals), and *A. triste* (1 adult individual) ticks. Lastly, a partial sequence of the *ompA* gene 100% (442/442 bp) identical to *R. rhipicephali* (KX434736) was found in *H. juxtakochi* (1 adult individual) (Table 2). Partial sequences of the *ompA* gene could not be obtained because of the low quality of amplified DNA. Thus, the partial *gltA* sequence from *A. cajennense* s.s. (one nymphal pool), *A. nodosum* (2 adult individuals) and *A. sculptum* (one nymphal pool) showed a 100% match (348/348 bp) to other SFG rickettsiae (KY753118, MK441839, MK720995, MK720994). In addition, we obtained partial *gltA* sequence 100% (326/326 bp) identical to *R. amblyommatis* (MH257786) in *A. sculptum* (1 nymphal pool) and *A. romitii* (1 adult individual). Lastly, sequences of the *gltA* gene found in *A. parvum* (1 adult individual) and *R. microplus* (1 adult individual) ticks were identical to each other and a 100% match (350/350 bp) to ‘*Candidatus* Rickettsia andeanae’ (MG887826), as shown in Table 2.

The GenBank nucleotide sequence accession numbers for the partial sequences generated in the present study are: OP823389, OP823390, OP823391, OP823392, and OP823393 for partial sequences of the *ompA* gene of *R. amblyommatis*, OP823395 and OP823396 of *R. parkeri,* OP823394 of *R. rhipicephali*; and OP823399 for partial sequences of the *gltA* gene of *Rickettsia* sp., OP823397 of *R. amblyommatis*, and OP484958 of ‘*Ca.* Rickettsia andeanae’.

## Discussion

This study revealed the presence of at least 21 tick species parasitizing wild animals in the states of Mato Grosso and Goiás, in the Central-West region of Brazil, between 2015 and 2018. The most abundant species among the collected ticks was the *A. cajennense* complex, followed by *A. dissimile*, *A. nodosum*, *R. microplus*, *A. scalpturatum*, *A. coelebs*, *A. dubitatum*, *A. rotundatum*, *A. romitii*, *A. calcaratum*, *R. linnaei*, *A. ovale*, *A. triste*, *A. humerale*, *A. oblongogutattum*, *A. longirostre*, *A. parvum*, *A. naponense*, *H. juxtakochi*, and *D. nitens*.

Amphibians and reptiles are the main hosts of all the parasitic stages of *A. dissimile* and *A. rotundatum* ticks (Nava et al., 2017; Alcantara et al., 2018; Luz et al., 2018; Torres et al., 2018). This was corroborated in the present study by the discovery of lizard and snake species with terrestrial or semiaquatic habits. However, this study identified new tick-host relationships with the amphibian *Rhinella diptycha* and nymphs of *A. rotundatum*. Additionally, the current record of adult *A. dissimile* ticks on *Eunectes notaeus* and *Paleosuchus palpebrosus* corresponds to a new host-parasite association. With regard to adult *A*. *humerale* ticks, our records of this species parasitizing *Chelonoidis denticulatus* and *Didelphis marsupialis* represent previously described associations (Ogrzewalska et al., 2010; Soares et al., 2015; Witter et al., 2016; Colle et al., 2020). Testudines (Testudinidae) are the common hosts for adult *A*. *humerale* ticks, while an infestation of Crocodylia and Mammalia is unexpected (Guglielmone et al., 2014), as is the infestation of the reptile *Phrynops geoffroanus* by adult *A. sculptum*, which was observed here for the first time. This is an unusual discovery since *A. sculptum* ticks are usually found parasitizing mammals (Nava et al., 2014; Martins et al., 2016).

As for information about tick species, the largest number of ticks comprising the *A. cajennense* species complex, represented in Brazil by *A. cajennense* s.s. and *A. sculptum* (the vector of *R. rickettsii*, agent of Brazilian Spotted Fever) (Szabó et al., 2013; Nava et al., 2014; Martins et al., 2016) was expected, given the large numbers of tapirs (n=14), capybaras (n=9) and giant anteaters (n=12), usual hosts for adults and immature stages of *A. sculptum* (Martins et al., 2016, 2023). Birds have been recorded as hosts of all the stages of *A. sculptum* in Brazil (Nava et al., 2017), as reported by Luz et al. (2016) in the state of Goiás and observed in this study for *A. sculptum* nymphs and adult ticks infesting *C. cristata* in the Cerrado biome. Similarly, the large number of adult *A. nodosum* ticks is presumably associated with the occurrence of anteaters (*Myrmecophaga tridactyla* and *Tamandua tetradactyla*), reported to be the main hosts of adult stages of *A. nodosum* (Nava et al., 2017). Although *A. cajennense* s.s. has been observed on a wide range of domestic and wild animals, including tapirs and giant anteaters (Martins et al., 2016; Luz et al., 2020), this paper describes the first tick-host relationship with *Mazama americana*.

*Rhipicephalus microplus* is strongly associated with cattle (Nava et al., 2017; Martins et al., 2023), and all the records of parasitism on *Subulo gouazoubira*, *Cerdocyon thous*, *Puma concolor*, and *Tapirus terrestris* described herein have already been reported previously by other authors. Even though horses are the main host of *D. nitens*, this parasite has been found in a variety of mammals, including wild carnivores (Labruna et al., 2005b; Guglielmone et al., 2014; Nava et al., 2017). In this paper, we describe for the first time, a *D. nitens* larva parasitizing *Chrysocyon brachyurus.* Therefore, tick infestations among livestock and horses should be considered accidental findings, possibly attributable to the fact that these animals share pastures with cattle and horses (Ramos et al., 2020).

In the order Carnivora, two new tick-host associations were observed, involving the species *A. cajennense* s.s. and *A. oblongoguttatum* nymphs found on wild cats *Puma concolor* and *Leopardus pardalis*, respectively. *Rhipicephalus linnaei*, recently recognized as belonging to the so-called “tropical lineage” of *Rhipicephalus sanguineus* s.l. (Šlapeta et al., 2022), is a species of the *R. sanguineus* complex commonly known as the brown dog tick. It has occasionally been found on mammals of different orders, with all parasitic stages of this tick strongly associated with domestic dogs (Nava et al., 2017), and here it was found parasitizing a rodent, the capybara *Hydrochoerus hydrochaeris*. This is probably due to these rodents’ population growth in urban and peri-urban areas, where *R. linnaei* is distributed throughout most of Brazil, particularly in these areas.

This study also found a new tick-host association of *A*. *cajennense* s.s. nymphs on a six-banded armadillo *Euphractus sexcinctus*. Although tapirs are considered the usual hosts of adults of *A*. *coelebs*, and the largest number of ticks found on one animal in our study was on a host parasitized by 14 specimens, adults and immature stages of this tick present low host specificity (Nava et al., 2017). The remaining tick-host associations described here have been previously reported.

The molecular survey indicated infection by an uncharacterized *Rickettsia* species belonging to the SFG in *A. cajennense* s.s., *A. nodosum*, and *A. sculptum*. Other studies have described uncharacterized *Rickettsia* in *A. humerale* (Colle et al., 2020) and *A. nodosum* (Lugarini et al., 2015). So, this report can indicate the possibility that other rickettsiae species not yet described should be infecting ticks from wild animals. Detection of *R. rhipicephali* in *H. juxtakochi* was expected since various studies carried out in the country have previously described this agent infecting this tick species (Labruna et al., 2005a, 2007; Soares et al., 2015). Although its pathogenic potential for humans is still unknown, experimental infections in mice suggest that it can cause moderately severe disease (Wikswo et al., 2008; Parola et al., 2013).

Another rickettsial agent found infecting *A. parvum*, and for the first time, *R. microplus* ticks, was ‘*Ca*. R. andeanae.’ Several studies have described infection by ‘*Ca*. R. andeanae’ in *Amblyomma* ticks, including *A. parvum* (Pacheco et al., 2007; Ogrzewalska et al., 2014; Nieri-Bastos et al., 2014; Lugarini et al., 2015; Maia et al., 2018), *A. auricularium* (Lugarini et al., 2015), *A. sculptum* (Witter et al., 2016), *A. maculatum* (Blair et al., 2004; Paddock et al., 2010; Flores-Mendoza et al., 2013), *A. pseudoconcolor* (Sebastian et al., 2022), *A. tigrinum* (Arrais et al., 2021), and *A. triste* (Abarca et al., 2012). The role of this bacterium as a human pathogen is still unknown (Ferrari et al., 2013); however, Krawczak et al. (2023) have proposed that high infection rates by ‘*Ca.* R. andeanae’ may favor the exclusion of other *Rickettsia* species in tick populations, as observed in their study of *R. parkeri* and *A. tigrinum* in southern Brazil.

Infection by *R. amblyommatis* in at least six tick species (*A. cajennense* s.s., *A. cajennense* s.l., *A. humerale*, *A. longirostre*, *A. sculptum*, *A. scalpturatum*, and *A. oblongoguttatum*) was expected, since this *Rickettsia* species has been reported in 34 tick species worldwide (Richardson et al., 2023). However, here we provide new data on *R. amblyommatis* infecting *A. romitii* and *A. nodosum* ticks. Considering the possibility of the genetic variability between the different strains of *R. amblyommatis* in South American (Sebastian et al., 2020), it is evident the importance of new data encompassing infection by this bacterium in two species of the genus *Amblyomma*, also contributing to a better understanding of ecological relationships involving ticks and agents transmitted by them in a wild environment. Despite the numerous reports of infection in ticks, the pathogenicity of *R. amblyommatis* to humans is still unknown. However, it is suspected that this may be a potential pathogen, in view of several serological reports of human infection, as well as a possible association with the occurrence of disease in some patients in the United States (Apperson et al., 2008; Delisle et al., 2016). Moreover, there is molecular evidence that this organism can infect dogs (Barrett et al., 2014), and cause fever and pathological signs of the disease in guinea pigs (Rivas et al., 2015).

Finally, molecular analyses revealed the presence of *R. parkeri* strain NOD infecting *A. scalpturatum* and *A. nodosum*, beyond *R. parkeri* strain Atlantic rainforest in *A. ovale*, and *R. parkeri* sensu stricto (s.s.) infecting *A. triste*. Phylogenetic analysis of *R. parkeri* indicated the existence of different strains: *R. parkeri* s.s., *R. parkeri* strain Atlantic rainforest, *R. parkeri* strain NOD, and *R. parkeri* strain Parvitarsum (Nieri-Bastos et al., 2018). In Brazil, *R*. *parkeri* strain Atlantic rainforest is responsible for mild cases of human rickettsiosis, transmitted mainly by adult *A. ovale* ticks and occurring widely throughout the Brazilian Atlantic Forest (Szabó et al., 2013; Barbieri et al., 2014; Krawczak et al., 2016; Moerbeck et al., 2016; Sevá et al., 2019).

*R. parkeri* s.s. has also been described infecting *A. triste* in southeastern and midwestern Brazil (Silveira et al., 2007; Widmer et al., 2011; Melo et al., 2015), and *A. tigrinum* (Weck et al., 2016) and *A. dubitatum* (Weck et al., 2017) in the Pampa biome. However, human infection by *R. parkeri* s.s. has never been confirmed in the laboratory in Brazil, although Weck et al. (2016) reported a possible human case transmitted by *A. tigrinum* in this country.

## Conclusions

This paper offers new information on tick-host associations with amphibians and reptiles, including nymphs of *A. rotundatum* parasitizing *R. diptycha*, adult *A. dissimile* ticks on *E. notaeus* and *P. palpebrosus*, and adult *A. sculptum* on *P. geoffroanus*. As for mammals, new associations were described for adults and nymphs of *A. cajennense* s.s. on *M. americana* and nymphs on *M. tridactyla*, larva of *D. nitens* on *C. brachyurus*, nymphal stages of *A. cajennense* s.s. and *A. oblongoguttatum* on *P. concolor* and *L. pardalis*, respectively, and lastly, the occurrence of *A*. *cajennense* s.s. nymphs on *E. sexcinctus*.

In addition, we have shown a wide diversity of rickettsiae infecting the tick fauna recorded, including a potentially human pathogen species, *R. parkeri*, demonstrating the importance of the study in the epidemiological context of One Health.
